# Opening the black box of *Anaplasma phagocytophilum* diversity: current situation and future perspectives

**DOI:** 10.3389/fcimb.2015.00061

**Published:** 2015-08-14

**Authors:** Thibaud Dugat, Anne-Claire Lagrée, Renaud Maillard, Henri-Jean Boulouis, Nadia Haddad

**Affiliations:** ^1^Laboratoire de Santé Animale, UMR Biologie Moléculaire et Immunologie Parasitaires, Agence Nationale de Sécurité Sanitaire de L'alimentation, de L'environnement et du Travail, Université Paris-EstParis, France; ^2^UMR Biologie Moléculaire et Immunologie Parasitaires, Ecole Nationale Vétérinaire d'Alfort, Université Paris-EstParis, France; ^3^Unité Pathologie des Ruminants, Ecole Nationale Vétérinaire de ToulouseToulouse, France

**Keywords:** *Anaplasma phagocytophilum*, diversity, epidemiology, granulocytic anaplasmosis, phylogeny, typing technique, tick-borne fever

## Abstract

*Anaplasma phagocytophilum* is a zoonotic obligate intracellular bacterium known to be transmitted by ticks belonging to the *Ixodes persulcatus* complex. This bacterium can infect several mammalian species, and is known to cause diseases with variable symptoms in many domestic animals. Specifically, it is the causative agent of tick-borne fever (TBF), a disease of important economic impact in European domestic ruminants, and human granulocytic anaplasmosis (HGA), an emerging zoonotic disease in Asia, USA and Europe. *A. phagocytophilum* epidemiological cycles are complex and involve different ecotypes, vectors, and mammalian host species. Moreover, the epidemiology of *A. phagocytophilum* infection differs greatly between Europe and the USA. These different epidemiological contexts are associated with considerable variations in bacterial strains. Until recently, few *A. phagocytophilum* molecular typing tools were available, generating difficulties in completely elucidating the epidemiological cycles of this bacterium. Over the last few years, many *A. phagocytophilum* typing techniques have been developed, permitting in-depth epidemiological exploration. Here, we review the current knowledge and future perspectives regarding *A. phagocytophilum* epidemiology and phylogeny, and then focus on the molecular typing tools available for studying *A. phagocytophilum* genetic diversity.

## Introduction

*Anaplasma phagocytophilum* is a tick-borne intragranulocytic alpha-proteobacterium, transmitted by hard ticks of the *Ixodes persulcatus* complex. It infects a large range of hosts worldwide, and is the causative agent of tick-borne fever (TBF) in ruminants (also known as bovine or ovine granulocytic anaplasmosis), and of human granulocytic anaplasmosis (HGA), two diseases which are becoming increasingly recognized in both Europe and the USA. *A. phagocytophilum* epidemiological cycles are complex, and involve different ecotypes circulating in various host species, as vector ticks feed on a large variety of vertebrates. To date, these epidemiological cycles are poorly understood, especially in Europe, as European reservoir hosts of the bacterium have not yet been identified.

In this review, we first present current knowledge concerning *A. phagocytophilum* phylogeny and epidemiology, with a particular focus on Europe and the USA where the vast majority of studies have been undertaken. We then review molecular typing tools that are currently available for studying *A. phagocytophilum*.

## Taxonomy

Veterinary medicine has been aware of the existence of *A. phagocytophilum* for decades. In 1932, during a study of louping-ill virus in sheep (*Ovis aries*) from Scotland, Gordon et al. discovered a new infectious disease caused by an unknown agent, but which was also transmitted by ticks (Gordon et al., 1932). The disease was named TBF, and in 1951 the causative agent was first described as *Rickettsia phagocytophila* (Foggie, 1951). However, the exact taxonomic position of *A. phagocytophilum* has been modified several times since 1951. The bacterium was reclassified as *Cytoecetes phagocytophila* in 1962 (Foggie, 1962), and as *Ehrlichia phagocytophila* in 1974 (Philip, 1974). Phylogenetic studies based on 16S RNA locus and *groESL* operon sequences drove Dumler et al. in 2001 to propose the unification of *E. phagocytophila, Ehrlichia equi* [first described in 1969 as the causative agent of equine granulocytic ehrlichiosis (Gribble, 1969)], and the agent of human granulocytic ehrlichiosis [first described in 1994 (Chen et al., 1994)] into one single species, *A. phagocytophilum* (Dumler et al., 2001). Using *gltA* gene sequences, Inokuma et al. also postulated that these three species were phylogenetically close, but did not question their placement within the *Ehrlichia* genus (Inokuma et al., 2001). In 2006 Dunning Hotopp et al. generated a phylogenetic tree based on the amino-acid sequences of 31 housekeeping proteins, which unified *A. phagocytophilum* and *Anaplasma marginale* into the same cluster, supporting the hypothesis of Dumler et al. (Dunning Hotopp et al., 2006). Even though their study provided strong arguments which supported the current classification of *A. phagocytophilum*, its taxonomy remains unclear, as only one *A. phagocytophilum* genome was compared. Considerable genetic variation has been consistently observed between different *A. phagocytophilum* strains (Barbet et al., 2013). For this reason, future phylogenomic analysis with the numerous *A. phagocytophilum* genomes (Dunning Hotopp et al., 2006; Barbet et al., 2013; Dugat et al., 2014b) and other newly available *Anaplasmataceae* genomic sequences will aid in refining the correct taxonomic position for *A. phagocytophilum*, possibly leading to further reclassification.

Because *A. phagocytophilum* has been reclassified several times since its discovery, the disease caused by this bacterium has multiple denominations: TBF, granulocytic ehrlichiosis, granulocytic anaplasmosis. In order to use a nomenclature common to all these diseases and to clearly designate the main host cell in which *A. phagocytophilum* multiplies, the most universal disease name will be used in this review: granulocytic anaplasmosis. For further clarification, the disease will be specified depending on the affected host species: HGA for humans, bovine granulocytic anaplasmosis (BGA) for cows (*Bos taurus*), ovine granulocytic anaplasmosis (OGA) for sheep, equine granulocytic anaplasmosis (EGA) for horses (*Equus caballus*) feline granulocytic anaplasmosis for cats (*Felis silvestris catus*), canine granulocytic anaplasmosis for dogs (*Canis lupus familiaris*).

## Granulocytic anaplasmosis symptoms

### GA symptoms in humans

HGA cases have been described throughout Asia, Europe, and North America (Jin et al., 2012; Ohashi et al., 2013; Kim et al., 2014). Clinical cases are both more serious and more frequent in the USA, where 2389 cases were reported by the Centers for Disease Control (CDC) in 2012 (Adams et al., 2014), as compared to the whole of Europe, where fewer than 100 confirmed cases have been described since its initial identification in Slovenia in 1997 (Petrovec et al., 1997). However, the number of reported European cases has risen over recent years, probably linked in part to improved surveillance (Cochez et al., 2011; Edouard et al., 2012). In Asia, HGA cases have been described in China, Japan, and South Korea (Ohashi et al., 2013; Zhang et al., 2013; Kim et al., 2014). In China, 46 confirmed and 16 probable cases were diagnosed from 2009 to 2010 with a 8.1% fatality rate (Zhang et al., 2013). However, seroprevalence studies suggest that the number of reported cases is underestimated in this country (Zhang et al., 2012, 2014).

HGA symptoms are variable and non-specific. Generally, patients present influenza-like symptoms with fever, headache, myalgia, and malaise (Ismail et al., 2010), and neurological symptoms have also been described (Ismail et al., 2010). Symptoms can be associated with decreased blood cell counts, thrombocytopenia, leukopenia, and anemia (Ismail et al., 2010). HGA can be fatal, but more than 60% of patients only present with moderate symptoms. However, HGA is not a benign disease, at least in the USA. In 2010, the CDC reported that 36% of HGA cases required hospitalization, of which 7% of these patients were referred to intensive care units (Centers for Disease Control and Prevention, Statistics and Epidemiology of Anaplasmosis)^1^.

A recent study reported severe clinical manifestations in Chinese patients, which have never been described to date in the USA and Europe: a multiple organ dysfunction syndrome (41.2%), gastrointestinal complications (22.6%), hemorrhagic complications (12.9%), a slow pulse (24.2%), facial edema (32.3%), and mental confusion (16.1%) (Zhang et al., 2013).

### GA symptoms in domestic ruminants

To date, confirmed GA cases in domestic ruminants have only ever been diagnosed in Europe. Even if *A. phagocytophilum* infection has been suggested by the presence of DNA and/or serological conversion in domestic ruminants from other continents, no actual clinical cases have been described outside Europe.

The most frequent GA symptoms in domestic ruminants are fever (> 41°C), weakness, anorexia, and abortion storms, occasionally resulting in death (Stuen et al., 2013a). In dairy cattle, a significant drop in milk production is a prominent clinical sign, and respiratory distress and leg edema have also been observed (Woldehiwet, 2006, 2010). As *A. phagocytophilum* also has immunosuppressive effects, secondary infection represents a common indication of GA in cattle herds (Woldehiwet, 2006, 2010). In addition, up to 30% of lambs (*O. aries*) infected with *A. phagocytophilum* developed tick pyaemia due to *Staphylococcus aureus* infection, which is considered the most common and serious GA complication in sheep. Pasteurellosis and septicemic listeriosis have also been described in this animal species (Woldehiwet, 2006).

Even though limited data has been collected in the field, it is commonly accepted that ruminant GA has an important economic impact in Europe. In the UK, approximately 300,000 lambs develop tick pyaemia annually, secondary to *A. phagocytophilum* infection. Most of them die or are subsequently of no economic value. Economic losses associated with cattle abortions and reduced milk production can also be significant (Brodie et al., 1986).

### GA symptoms in other domestic animals

In horses, EGA cases have been reported in both the USA and Europe. Many symptoms are similar to those observed in humans, and include fever, weakness, anorexia, ataxia, icterus leg edemas, thrombocytopenia, anemia, and leucopenia (Bermann et al., 2002; Butler et al., 2008; Jahn et al., 2010). Neurological symptoms have also been reported (Gussmann et al., 2014).

In dogs, malaise, lethargy, fever, inappetence or anorexia, weakness, indisposition, lymphadenomegaly, hepatomegaly, and splenomegaly have all been described (Carrade et al., 2009; Silveira et al., 2015).

In cats, a recent study reported lethargy in all animals, and fever and anorexia in 15/16 studied cats (Savidge et al., 2015). Hepatosplenomegaly, ataxia, conjunctivitis, and elevation of the nictitating membranes were also observed in some animals, associated with different blood cell count disorders, such as lymphopenia (7/11 cats), thrombocytopenia (7/11), neutropenia (3/11), and leukopenia (2/11) (Savidge et al., 2015).

## *A. phagocytophilum* epidemiological cycles

As Stuen et al. reviewed the distribution of *A. phagocytophilum* according to continents and host species in 2013 (Stuen et al., 2013a), our review will first focus on the roles of the different actors involved in *A. phagocytophilum* epidemiological cycles, and then on the spread of *A. phagocytophilum* infection in both hosts and populations.

### One bacterium, but multiple variants

The *A. phagocytophilum* species can be divided into several genetic variants^2^ likely involved in different epidemiological cycles. Here we will describe the most studied variants defined using the nucleotide sequence of the 16S RNA locus. Variants defined using other loci will be discussed in the paragraph “Tools for studying the genetic diversity of *A. phagocytophilum*.”

In the USA, variations in the 16S RNA locus distinguishes at least two major variants: Ap-V1 and Ap-ha (Chen et al., 1994). Other 16S RNA variants have also been detected, but as few epidemiological and biological data are currently available concerning these variants (Belongia et al., 1997; Massung et al., 2002; Michalski et al., 2006), we will focus on the major variants, Ap-V1 and Ap-ha. These two variants can coexist in the same geographical areas, and could also be transmitted by the same vectors (Courtney et al., 2003). These observations suggest that Ap-V1 and Ap-ha do not segregate according to geography, but rather according to the vertebrate hosts that they can or cannot infect. Indeed, the white-tailed deer (*Odocoileus virginianus*) is a major reservoir host for Ap-V1, but not Ap-ha (Massung et al., 2005; Reichard et al., 2009). Conversely, the white-footed mouse (*Peromyscus leucopus)* is a principle reservoir host for Ap-ha, but not Ap-V1 (Massung et al., 2003). Moreover, Ap-ha is pathogenic for humans, dogs and horses, whereas Ap-V1 has never been implicated in human infections. The tropism observed at the host level can also be observed at the cellular level: whereas Ap-ha can infect and multiply in both *Ixodes* (ISE6, IDE8) (Munderloh et al., 1999) and human (HL-60) cell lines (Goodman et al., 1996; Horowitz et al., 1998), Ap-V1 has only been cultivated in *Ixodes* cell lines (Woldehiwet et al., 2002; Massung et al., 2007).

*A. phagocytophilum* epidemiology in Europe is poorly understood compared to the USA. In contrast to what has been observed in the USA, European variants carrying 16S RNA sequences which correspond to both Ap-V1 and Ap-ha variants have been detected in several wild ruminant species.

### Vectors

Granulocytic anaplasmosis is not a contagious disease, as *A. phagocytophilum* is only transmitted by vectors^3^. Known arthropod vectors of *A. phagocytophilum* are ticks belonging to the *Ixodes* genus: *I. ricinus* in Europe, *I. scapularis* in Eastern USA, *I. pacificus* and *I. spinipalpis* in Western USA, and *I. persulcatus* in Asia and Russia (MacLeod and Gordon, 1933; Telford et al., 1996; Des Vignes et al., 1999; Zeidner et al., 2000; Burkot et al., 2001; Stuen et al., 2013a). *A. phagocytophilum* DNA has also been detected in many other tick genera and species, but their vector competence has not yet been proven (Stuen et al., 2013a). Some other *Ixodes* species, such as *I. trianguliceps* (Bown et al., 2003, 2008, 2009) or *I. hexagonus* (Silaghi et al., 2012a) appear to be involved in epidemiological cycles distinct from those involving *I. ricinus*. This will be further discussed in the next section. Moreover, *A. phagocytophilum* DNA has been detected in areas where *I. ricinus* is rare or even absent. For example, several elements suggest that *Rhipicephalus* sp could be the vector involved in *A. phagocytophilum* transmission in the French Camargue region (Leblond et al., 2005; Moulin, 2009; Chastagner et al., 2013; Dugat et al., 2014a). However, as the vector competence of these ticks is unknown, the next paragraph focuses on confirmed *A. phagocytophilum* vectors.

*Ixodes spp* are telotropic species, meaning that they are able to feed on a large variety of host species (Stanek, 2009). True trophic “preferences” are difficult to identify, and are currently under discussions. Nevertheless, it has been suggested that larvae preferentially parasitize small mammals (such as rodents), nymphs parasitize bigger mammals (such as rabbits), and adult ticks target large mammals (such as wild and domestic ruminants) (Chauvet, 2004; Sonenshine and Roe, 2014).

The presence of *Ixodes spp* within a territory mainly depends on climatic conditions (between 10 and 30°C, and >80% relative humidity) and on the availability of hosts. In Europe, *I. ricinus* territories have expanded over the past few years (Materna et al., 2005; Jore et al., 2011; Jaenson et al., 2012), probably due to climatic and ecological modifications linked to both anthropic factors (Medlock et al., 2013; Jore et al., 2014) and forest extension. The low host specificity of *I. ricinus*, and its tolerance to various environments, can explain its broad spread across Europe.

Ticks belonging to the *Ixodes* genus have a three-host triphasic lifecycle. Each stage requires one blood meal (except for males which do not feed) before moving to the next stage. Ticks may acquire *A. phagocytophilum* while feeding on an infected animal host or through transstadial transmission (from one stage to the next). For *Ixodes* species, transovarial transmission (from the female adult to the eggs) does not occur according to current knowledge. *Two* experimental studies have reported *A. phagocytophilum* transmission between ticks via co-feeding^4^, without the need for an infected animal, but this mechanism appears to be rare (Levin and Fish, 2000; Ogden et al., 2003). Infected ticks can then transmit *A. phagocytophilum* to new hosts during the blood meal of its following stage. To summarize, *Ixodes spp* can be infected by *A. phagocytophilum* at each stage (except as eggs), but only nymphs and adult females can transmit this bacterium. For this reason, and because transovarial transmission has never been described in these ticks, *Ixodes spp* are unable to support persistent *A. phagocytophilum* infection, and therefore cannot be considered as reservoir hosts for this pathogen.

### Reservoir and/or spillover hosts

A reservoir is a biotic or abiotic environment which permits a pathogen to persist in a sustainable manner, under given conditions. As *A. phagocytophilum* is an obligate intracellular bacterium, its reservoirs should be animal hosts which permit its survival, particularly (but not exclusively) outside the activity period of its vectors. For tick-borne pathogens, if the microorganism is transovary transmitted, the tick can also be a reservoir host, as has been demonstrated for *Rickettsia helvetica* and for the tick-borne encephalitis virus in *I. ricinus* (Nuttall and Labuda, 2003; Sprong et al., 2009; Rizzoli et al., 2014). As mentioned above for *A. phagocytophilum*, such transmission has never been described in *Ixodes* spp, indicating that they are not reservoir hosts for this bacterium. *A. phagocytophilum* transovarial transmission has been shown to occur at high frequency (up to 40%) for *Dermacentor albipictus* in the USA. However, under experimental conditions, infected F1 larval ticks which were reared to maturity failed to transmit the bacteria to their F2 larval progeny (Baldridge et al., 2009). Thus, even though *A. phagocytophilum* transovarial transmission in *D. albipictus* has been proven, its impact on bacterial persistence remains highly questionable. Therefore, according to currently available data, we assume that *A. phagocytophilum* reservoir hosts must be vertebrate organisms.

For some wild species, their precise role in *A. phagocytophilum* epidemiological cycles remains to be determined. Some species, rather than being reservoir hosts, could be spillover hosts, thus facilitating the transmission of *A. phagocytophilum* between reservoir hosts and susceptible hosts, particularly but not only when contacts between these categories of hosts are infrequent. As *A. phagocytophilum* epidemiological cycles are complex and not completely elucidated, it is important to keep in mind that some of the host species listed below that are supposed or suspected to be reservoir hosts, could in fact be spillover hosts.

#### Wild ruminants

In the USA, the white-tailed deer is the main reservoir host for *A. phagocytophilum* variant Ap-V1. The role of other wild ruminant species has not been investigated in depth to date. In California, one study has reported high infection prevalence^5^ in mule deer (*Odocoileus hemonius hemonius*) and Tule elks (*Cervus elaphus nannodes*) (Foley et al., 1998), suggesting that these two wild ruminants could also be reservoir hosts for *A. phagocytophilum*.

In Europe, *A. phagocytophilum* DNA has been detected in many wild ruminant species. In particular, red deer (*Cervus elaphus*) and roe deer (*Capreolus capreolus*) have been suspected to be *A. phagocytophilum* reservoir hosts (Alberdi et al., 2000) for several reasons. Firstly, *A. phagocytophilum* infection can persist for up to 3 months in red deer (Stuen et al., 2001). Secondly, both red and roe deer have high prevalences of *A. phagocytophilum* infection, as indicated by the presence of *A. phagocytophilum* DNA: up to 87.5 and 98.9% for red and roe deer, respectively (Stuen et al., 2013a). However, several recent studies strongly suggest that roe deer are not reservoir hosts for human, dog, or horse variants, nor for domestic ruminant variants (Rymaszewska, 2008; Scharf et al., 2011; Chastagner et al., 2014; Dugat et al., 2014a; Huhn et al., 2014). For this reason, we and other authors have hypothesized that roe deer could be reservoir hosts for their own specific *A. phagocytophilum* variants. Conversely, further data also indicate that red deer could be reservoir hosts for domestic ruminant variants (Petrovec et al., 2002; Majazki et al., 2013; Chastagner et al., 2014; Dugat et al., 2014a; Huhn et al., 2014). However, in contrast to what has been previously proposed (Rymaszewska, 2008), red deer are not likely to be reservoir hosts for human, dog, or horse variants (Chastagner et al., 2014; Dugat et al., 2014a; Huhn et al., 2014).

*A. phagocytophilum* is also highly prevalent in other European wild ruminants, such as elks (*Alces alces*), alpine ibex (*Capra ibex*), sika deer (*Cervus Nippon*), fallow deer (*Dama dama*), and chamois (*Rupicapra rupicapra*). However, the role of these animals in *A. phagocytophilum* epidemiological cycles has not yet been clarified.

In Asia, *A. phagocytophilum* DNA has been detected at high prevalence rates in Korean water deer (*Hydropotes inermis argyropus*) and in sika deer (*C. nippon*) up to 46 and 63.6%, respectively (Stuen et al., 2013a), suggesting that these animals could be reservoir hosts. Two studies reported that the variants obtained from sika deer in Japan are genetically distant from those infecting wild ruminants and other animal species in Asia, Europe and the USA (Kawahara et al., 2006; Masuzawa et al., 2011). However, the number of studies that have been conducted on wild ruminants in Asia is too limited to draw any definitive conclusion about the role played by these animals in *A. phagocytophilum* epidemiological cycles.

#### Rodents

Rodents are major hosts for ticks, mainly the larval and nymph stages. In the USA, *A. phagocytophilum* infection prevalence varies from 1.8 to 88.4%, depending on the study and the rodent species (Stuen et al., 2013a). In the eastern part of the country, the white-footed mouse, and possibly the gray squirrel (*Sciurus carolinensis*), are reservoir hosts for the Ap-ha variant (Levin et al., 2002; Massung et al., 2003). Dusky-footed woodrats (*Neotoma fuscipes*) and yellow-cheeked chipmunks (*Tamias ochrogenys*) are also considered as reservoir hosts for this variant, particularly in the western part of the USA, from where the white-footed mouse is absent (Nicholson et al., 1999; Levin et al., 2002; Foley et al., 2008, 2011; Nieto and Foley, 2009). Finally, in Colorado, the high prevalence of *A. phagocytophilum* infection observed in deer mouse (*Peromyscus maniculatus*) and in mexican woodrat (*Neotoma mexicana*), have led some authors to conclude that these rodents are reservoir hosts for this pathogen. They also suggested that these animals could be involved in an alternative epidemiological cycle, in which *I. spinipalpis* could be the vector (Zeidner et al., 2000).

In Europe, *A. phagocytophilum* DNA has been detected in at least nine different rodent species (Stuen et al., 2013a). However, the prevalence of *A. phagocytophilum* infection is lower in rodents than in wild ruminants (Barandika et al., 2007; Silaghi et al., 2012b; Stuen et al., 2013a; Baráková et al., 2014). Phylogenetic analysis based on *groEL* (Jahfari et al., 2014), *msp4* (Baráková et al., 2014) and *ankA* (Majazki et al., 2013) loci have revealed that rodent strains belong to a different cluster than other *A. phagocytophilum* strains, making it unlikely that these rodent strains circulate in *A. phagocytophilum* epidemiological cycles involving other mammals. According to recent studies, rodents could be *A. phagocytophilum* reservoir hosts in an independent epidemiological cycle, involving only rodents as mammalian hosts (Bown et al., 2009; Blaòarová et al., 2014). In the UK and Central Europe, at least two independent epidemiological cycles have been described: the first may involve rodents as reservoir hosts and *I. trianguliceps* as vectors, whereas the second might involve wild ruminants as reservoir hosts and *I. ricinus* as vectors (Bown et al., 2003, 2008, 2009; Blaòarová et al., 2014).

In Asia, *A. phagocytophilum* DNA has been detected in at least 16 different rodent species (Stuen et al., 2013a). High prevalences of infection have been observed in several species in this continent-up to 55.5 and 23.6% in brown house rats (*Rattus norvegicus*) and black-striped field mice (*Apodemus agrarius*), respectively (Stuen et al., 2013a)-suggesting that they could be reservoir hosts for *A. phagocytophilum*. In particular, due to its high rate of infection and its wide geographical distribution in Asia, some authors consider that the black-striped field mice is the most important reservoir of *A. phagocytophilum* in this continent (Zhan et al., 2010; Jin et al., 2012; Yang et al., 2013). However, its reservoir competence has not been yet experimentally demonstrated (Zhan et al., 2008); thus, more studies are needed before drawing any definitive conclusion regarding the role of rodents as reservoir hosts of *A. phagocytophilum* in Asia.

#### Other wild species

*A. phagocytophilum* DNA has been detected in 24.6% of raccoons (*Procyon lotor*) tested in Connecticut (Levin et al., 2002). Raccoons can be experimentally infected by both Ap-ha and Ap-V1 variants, but only appear to be reservoir hosts for Ap-ha (Levin et al., 2002; Yabsley et al., 2008).

On Nantucket island (Massachusetts), the cottontail rabbit (*Sylvilagus floridanus*) could be involved in an alternative epidemiological cycle as a reservoir host, with *Ixodes dentatus* as the vector (Goethert and Telford, 2003).

In Europe, hedgehogs (*Erinaceus europaeus*) and wild boars (*Sus scrofa*) are also suspected to be reservoir hosts for human *A. phagocytophilum* variants for the following reasons (Skuballa et al., 2010; Silaghi et al., 2012a, 2014; Huhn et al., 2014). Firstly, high *A. phagocytophilum* infection prevalence has been observed in these animals (up to 85.4 and 12%, respectively) (Stuen et al., 2013a). Moreover, variants infecting hedgehogs and wild boars have identical 16S RNA sequences to those variants infecting humans (Silaghi et al., 2012a, 2014). In addition, a recent MLST study has revealed that variants infecting hedgehogs, wild boars and humans cluster together (Huhn et al., 2014). However, the immune system of wild boars quickly eliminates their infection (Galindo et al., 2012) and infection duration in hedgehogs also appears to be short (Silaghi et al., 2012a). Thus, their role as reservoir hosts has been recently questioned (Galindo et al., 2012; Silaghi et al., 2012a).

Hedgehogs could also be involved in an alternative epidemiological cycle in which *I. hexagonus* could be the vector (Silaghi et al., 2012a). Hedgehogs are often heavily parasitized by ticks, particularly *I. hexagonus* and *I. ricinus*, (Pfäffle et al., 2011; Silaghi et al., 2012a). Silaghi *et al*. have shown that hedgehogs, as well as the *I. ricinus* and *I. hexagonus* that feed on them, are all infected with *A. phagocytophilum* (Silaghi et al., 2012a). All variants reported in that study were able to infect both *Ixodes* species and hedgehogs (Skuballa et al., 2010; Silaghi et al., 2012a), but one variant (human pathogenic variant A) was more often associated with hedgehogs and *I. ricinus* (Silaghi et al., 2012a). Although the 16S RNA locus is not a highly discriminatory marker, this study suggested that variants infecting *I. hexagonus* could also infect *I. ricinus*, therefore the epidemiological cycles involving these two vectors may be interconnected. Moreover, the coexistence of variants involved in different epidemiological cycles in these hedgehogs could lead to genetic recombinations between variants, contributing to *A. phagocytophilum* genetic diversity. If this hypothesis of interconnected epidemiological cycles is validated, the hedgehog situation could be completely different from the independent epidemiological cycles described in UK rodents.

To date, only a few groups have studied the role of birds in *A. phagocytophilum* epidemiological cycles (Stuen et al., 2013a). One such study did detect high levels of *A. phagocytophilum* DNA in several different bird species. However, only a few birds from each species were tested (often only one), and for this reason it is difficult to conclude if birds play a significant role in *A. phagocytophilum* epidemiological cycles (De la Fuente et al., 2005). Contradictorily, a second study reported that only 1/11 common blackbirds (*Turdus merula*), was infected with *A. phagocytophilum*, and that no infection was observed in any of the eight other bird species examined. In this study, the authors used *groEL* sequences to demonstrate that birds and bird-fed ticks harbored specific *A. phagocytophilum* variants, suggesting that these animals could be involved in an independent avian epidemiological cycle (Jahfari et al., 2014). Studying tick feeding on an animal species can provide indirect clues as to its role in a pathogen's epidemiological cycle. Prevalence of *A. phagocytophilum* infection in bird-fed ticks is low, suggesting that birds are unlikely to be reservoir hosts (Skotarczak et al., 2006; Hildebrandt et al., 2010; Palomar et al., 2012; Geller et al., 2013; Lommano et al., 2014). Nevertheless, migrating birds can travel long distances and could be important for the spread of *A. phagocytophilum* and its vector(s) (Hildebrandt et al., 2010; Palomar et al., 2012; Dingler et al., 2014).

#### Domestic ruminants

In Europe, sheep are one of the main hosts of *A. phagocytophilum*. Intra-herd infection prevalence can reach up to 37% (Norway) (Stuen et al., 2013b), and at least 300,000 OGA cases are diagnosed every year in both Norway and the UK (Woldehiwet, 2006). During experimental infection of sheep, *A. phagocytophilum* DNA was detected by qPCR at 358 days post-infection. The authors did not confirm the presence of viable bacteria, yet this result suggests that sheep can maintain *A. phagocytophilum* infection for at least a year (Thomas et al., 2012). Thus, even if sheep develop OGA symptoms during the acute phase of infection, they could potentially also be reservoir hosts for their own *A. phagocytophilum*, as they can maintain constant infection with certain periods of high bacteremia. This dual role could contribute to the high number of OGA cases observed every year in some European countries.

### Conclusions: hypothetical epidemiological cycles of *A. phagocytophilum*

#### In the USA (Figure 1, Table 1)

**Figure 1:**
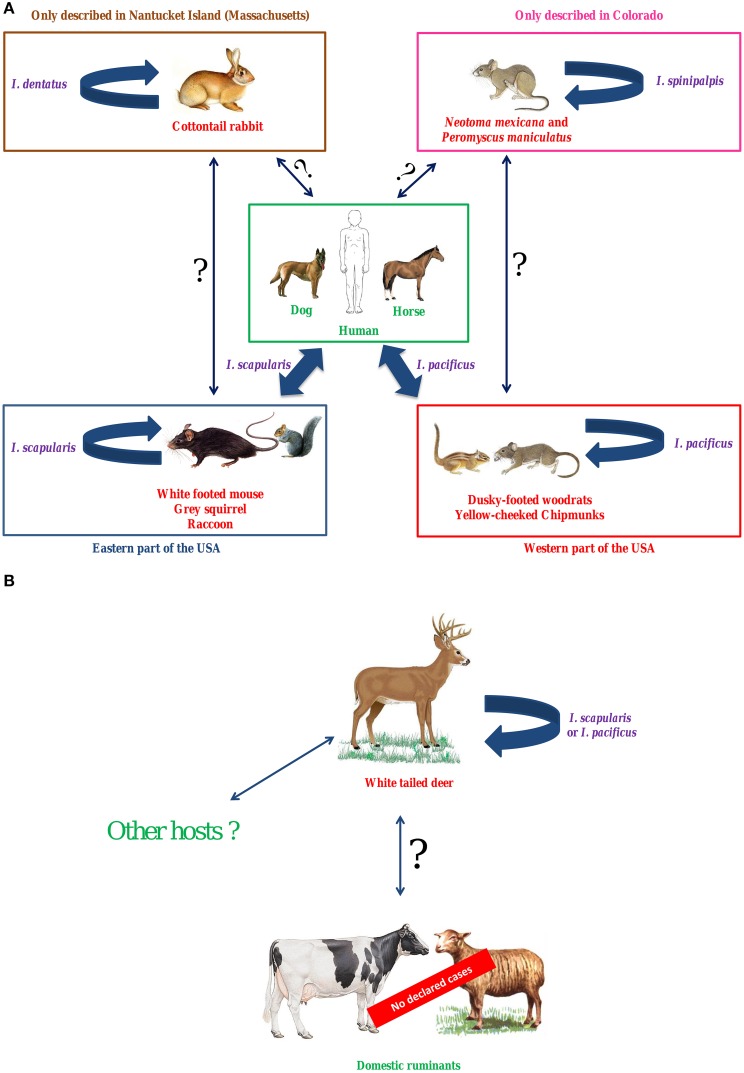
**Hypothetical epidemiological cycles of ***A. phagocytophilum*** variants Ap-ha (A) and Ap-V1 (B) in the USA**. In the USA, *I. scapularis* (Eastern states) and *I. pacificus* (Western states) are the main vectors of *A. phagocytophilum*. To date, two predominant independent epidemiological cycles, involving the two different variants Ap-ha and Ap-V1, have been identified in this country. White-footed mice, raccoons, gray squirrels (Eastern USA) and other rodents (Western USA) are reservoir hosts for the first variant, Ap-ha, which also infects humans, dogs, and horses. Variant Ap-V1 circulates in a secondary epidemiological cycle, which involves white-tailed deer as reservoir hosts. Domestic ruminants can be experimentally infected by this variant, however to date, no BGA cases have been reported in the USA. Thus, under natural conditions, variant Ap-ha does not appear capable of infecting ruminants, whereas variant Ap-V1 cannot infect either rodents or humans. Finally, two potential alternative *A. phagocytophilum* epidemiological cycles have been described in the USA: the first involves *N. mexicana* and *P. maniculatus* as reservoir hosts and the tick *I. spinipalpis* as vector, whereas the second involves the cottontail rabbit as a reservoir host and *I. dentatus* as vector. In purple, vectors; in red, reservoir hosts; in green, dead-end hosts (or clinical hosts); Large solid arrow, demonstrated transmission; Solid arrow with question mark, unknown transmission.

In the USA, *I. scapularis* (Eastern states) and *I. pacificus* (Western states) are the main vectors of *A. phagocytophilum*. To date, two predominant independent epidemiological cycles, involving the two different variants Ap-ha and Ap-V1, have been identified in this country. White-footed mice, raccoons, gray squirrels (Eastern USA), and other rodents (Western USA) are reservoir hosts for the first variant, Ap-ha, which also infects humans, dogs and horses (Figure 1A). Variant Ap-V1 circulates in a secondary epidemiological cycle, which involves white-tailed deer as reservoir hosts (Figure 1B). Domestic ruminants can be experimentally infected by this variant, however to date, no BGA cases have been reported in the USA. Thus, under natural conditions, variant Ap-ha does not appear capable of infecting ruminants, whereas variant Ap-V1 cannot infect either rodents or humans. Finally, two potential alternative *A. phagocytophilum* epidemiological cycles have been described in the USA: the first involves *N. mexicana* and *P. maniculatus* as reservoir hosts and the tick *I. spinipalpis* as vector, whereas the second involves the cottontail rabbit as a reservoir host and *I. dentatus* as vector.

**Table 1:** **Main variants, reservoir hosts, and vectors of ***A. phagocytophilum*** in the USA**.

**Variant**	**Reservoir host(s)**	**Vector(s)**	**Geographical area**	**Reference(s)**
Ap-ha	Cottontail rabbit (*S. floridanus*)	*I. dentatus*	Nantucket Island (Massachusetts)	Goethert and Telford, 2003
	Mexican woodrat (*N. mexicana*)Deer mouse (*P. maniculatus*)	*I. spinipalpis*	Colorado	Zeidner et al., 2000
	White-footed mouse (*P. leucopus*)Gray squirrel (*S. carolinensis*)Raccoon (*P. lotor*)	*I. scapularis*	Eastern USA	Levin et al., 2002; Massung et al., 2003
	Dusky-footed woodrats (*N. fuscipes*)Yellow-cheeked chipmunks (*T. ochrogenys*)	*I. pacificus*	Western USA	Nicholson et al., 1999; Levin et al., 2002; Foley et al., 2008, 2011; Nieto and Foley, 2009
Ap-V1	White-tailed deer (*Odocoileus virginianus*)	*I. scapularis* *I. pacificus*	Probably the whole country	Massung et al., 2005; Reichard et al., 2009

#### In Europe (Figure 2, Table 2)

**Figure 2:**
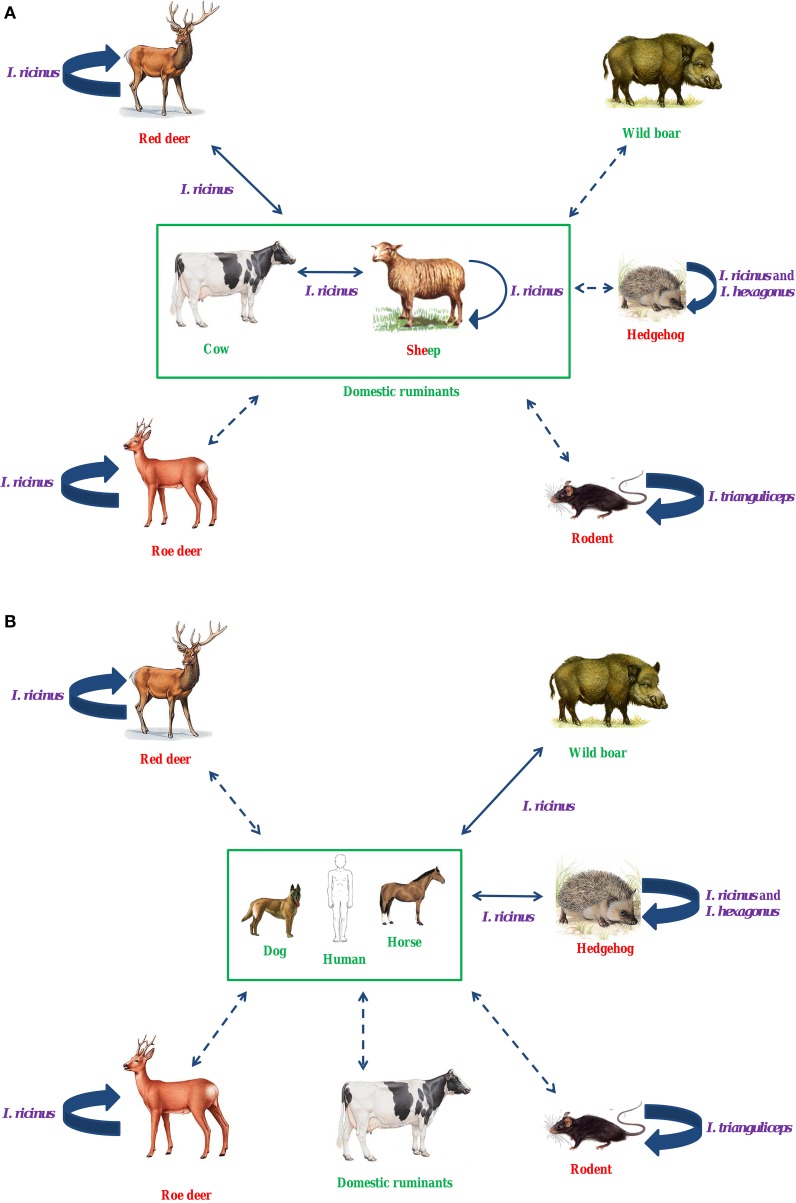
**Hypothetical epidemiological cycles of ***A. phagocytophilum*** in ruminants (A) and in humans, dogs, and horses (B) in Europe**. *I. ricinus* is the main European vector of *A. phagocytophilum*. Several studies suggest that red deer could be reservoir hosts for domestic ruminant variants. Roe deer are unlikely to be reservoir hosts for human, horse or pet variants, nor for domestic ruminant variants. Roe deer may be involved in another epidemiological cycle, and could maintain their “own” specific variant(s). Hedgehogs have been suspected as reservoir hosts for human variants, but this remains unproven. Similar to roe deer, hedgehogs could also be involved in an alternative epidemiological cycle, in which *I. hexagonus* could be the vector. Different rodent species could be involved in an independent epidemiological cycle involving *I. trianguliceps* as vector. In purple, vectors; in red, reservoir hosts; in green, dead-end hosts (or clinical hosts); Large solid arrow, known transmission; Solid arrow, unknown, but possible transmission; Dotted arrow, unknown, but unexpected transmission.

*I. ricinus* is the main European vector of *A. phagocytophilum*, but to date, reservoir hosts for this bacterium have not been clearly identified. However, several studies suggest that red deer could be reservoir hosts for domestic ruminant variants (Figure 2A). Roe deer are unlikely to be reservoir hosts for human, horse or pet variants, nor for domestic ruminant variants (Figure 2). Roe deer may be involved in another epidemiological cycle, and could maintain their “own” specific variant(s). Hedgehogs have been suspected as reservoir hosts for human variants, but this remains unproven (Figure 2B). Similar to roe deer, hedgehogs could also be involved in an alternative epidemiological cycle, in which *I. hexagonus* could be the vector. Different rodent species could be involved in an independent epidemiological cycle involving *I. trianguliceps* as vector.

**Table 2:** **Major theoretical reservoir hosts, dead-end hosts, and vectors of ***A. phagocytophilum*** in Europe**.

**Theoretical reservoir host**	**Vector(s)**	**Theoretical dead-end host(s)**	**Geographical area**	**Reference(s)**
Roe deer (*C. capreolus*)	*I. ricinus*	Unknown	Europe	Alberdi et al., 2000; Rymaszewska, 2008; Scharf et al., 2011; Chastagner et al., 2014; Dugat et al., 2014a; Huhn et al., 2014
Red deer (*C. elaphus*)	*I. ricinus*	Dometic ruminants	Europe	Stuen et al., 2001; Petrovec et al., 2002; Majazki et al., 2013; Chastagner et al., 2014; Dugat et al., 2014a; Huhn et al., 2014
Hedgehog (*E. europaeus*)	*I. ricinus* and *I. hexagonus*	Human	Germany probably other countries	Skuballa et al., 2010; Pfäffle et al., 2011; Silaghi et al., 2012a; Huhn et al., 2014
Different rodent species	*I. trianguliceps*	Unknown	United Kingdom and Slovakia	Bown et al., 2008, 2009; Majazki et al., 2013; Baráková et al., 2014; Blaòarová et al., 2014; Jahfari et al., 2014
Sheep (*O. aries*)	*I. ricinus*	Dometic ruminants	Europe	Thomas et al., 2012; Stuen et al., 2013b

## Dynamics of the infection

### *A. phagocytophilum* transmission between vertebrate and invertebrate hosts

#### *A. phagocytophilum* transmission from the vector to the vertebrate host

Experimental transmission of *A. phagocytophilum* via *I. scapularis* to mice occurs from between 24 and 48 h after tick attachment (Hodzic et al., 1998; des Vignes et al., 2001). The median infectious dose at which *morulae* are observed in mice is between 10^4^ and 10^5^ *A. phagocytophilum* cells (Hodzic et al., 1998). In sheep, intravenously injecting an average of 1.3 infected neutrophil polynuclear cells was sufficient to induce TBF symptoms, but initial bacterial number per polynuclear cell was not assessed (Stuen and Artursson, 2000). As the very processes of tick attachment and feeding stimulate *A. phagocytophilum* multiplication, it is not possible to infer naturally occurring infection rates from these experimental conditions (Hodzic et al., 1998, 2001; Foley and Nieto, 2007). Additionally, incubation period is inversely dose-dependent: the higher the infectious dose, the shorter the incubation period (Stuen and Artursson, 2000), but according to Stuen et al., infectious dose is not correlated with severity of symptoms (Stuen and Artursson, 2000).

#### *A. phagocytophilum* transmission from vertebrate host to vector

Experimental *A. phagocytophilum* transmission from infected mice to *I. scapularis* occurs between 24 and 48 h after the initiation of blood meals (Hodzic et al., 1998). Subsequently *A. phagocytophilum* migrates to the gut and the salivary glands of the tick, with positive detection at these locations from 24 h after the start of the blood meal (Sukumaran et al., 2006).

The probability of *A. phagocytophilum* being transmitted from an infected sheep to *I. ricinus* is highest during the acute phase of infection, which then decreases with time as persistent infection is established and bacteremia decreases (post-acute phase) (Ogden et al., 2003). The risk of transmission also increases with the number of ticks parasitizing the animal (Ogden et al., 2003), and nymphs also have a higher likelihood of acquiring *A. phagocytophilum* than larvae (Ogden et al., 2003).

In order to more efficiently control GA, it is important to understand the dynamics of *A. phagocytophilum* infection, but this is generally an aspect that has been poorly studied. However, some important data have been obtained using sheep and mouse models. Nevertheless, as various *A. phagocytophilum* ecotypes appear to circulate in differing epidemiological cycles, the results presented here cannot be generalized across all species.

### Infection spread within vertebrate host populations

*A. phagocytophilum* spread within host populations depends of the presence of: (1) the bacteria itself; (2) *A. phagocytophilum*-receptive hosts; (3) *A. phagocytophilum*-susceptible hosts; (4) competent vectors for *A. phagocytophilum*; and (5) the ability of these vectors to transmit *A. phagocytophilum*. In addition, increases in host and/or vector population size and/or their distribution areas can promote the geographical spread of the bacterium.

Roe deer can have high prevalence levels of *A. phagocytophilum* infection (Stuen et al., 2013a), and are often parasitized by many ticks (Vor et al., 2010). Deer can travel long distances (Vor et al., 2010), aiding the spread of both the bacteria and its vector(s) (Overzier et al., 2013). The same phenomenon could also occur with migratory birds (Alekseev et al., 2001; Bjöersdorff et al., 2001). However, the very low prevalence of *A. phagocytophilum* infection in such birds (Stuen et al., 2013a) suggests that their involvement in *A. phagocytophilum* expansion is limited.

*A. phagocytophilum* transplacental transmission has been described under natural conditions in cows (Henniger et al., 2013), and observed under experimental conditions in sheep (Reppert et al., 2013). However, evaluating the role of this transmission route in the spread of *A. phagocytophilum* infection has been difficult due to lack of data. In France, one study reported the presence of *A. phagocytophilum* DNA in 52/84 aborted bovine fetuses (5.2%), and in 484 placental swabs or 357 vaginal swabs from cows which had aborted (Guatteo et al., 2014). The presence of DNA alone does not conclusively confirm either the existence of viable bacteria, or their direct involvement in abortions, therefore additional studies are needed before drawing any definitive conclusions. Several human cases of *A. phagocytophilum* transmission occurring *via* blood transfusion have also been reported (Annen et al., 2012; Alhumaidan et al., 2013; Townsend et al., 2014). Finally, Zhang et al., reported nosocomial transmission of HGA in a Chinese hospital, through direct contact with blood or respiratory secretions (Zhang et al., 2008).

To summarize this section, many gaps still exist in current *A. phagocytophilum* epidemiology knowledge. This could be at least partly due to the fact that until very recently, few effective typing techniques were available. The second part of this review will be devoted to describing these tools.

## Molecular tools for studying *A. phagocytophilum*

Historically, phenotypic approaches (such as serotyping) were used to study bacterial diversity. Apart from a study performed by Inokuma et al. (2003), *A. phagocytophilum* serotyping methods have rapidly been replaced by more efficient molecular biology-based typing methods.

### *A. phagocytophilum* genome features

The *A. phagocytophilum* genome is a double-stranded chromosome from 1.47 to 1.48 Mb (only fully sequenced genomes were considered here) without any associated plasmids (Dunning Hotopp et al., 2006; Barbet et al., 2013). Complete sequenced genomes contain between 1140 and 1411 genes (including protein coding sequences, rRNA, tRNA, and pseudogenes) (Dunning Hotopp et al., 2006; Barbet et al., 2013; Dugat et al., 2014b). The HZ genome reference strain encodes 1369 proteins, of which 747 (55%) had known functions at the time of sequencing (Dunning Hotopp et al., 2006). Following infection of HL-60 cells, the expression of 1212 proteins was detected, corresponding to 89.3% of predicted proteins from the HZ strain genome (Lin et al., 2011). The vast majority of proteins with known function (99%) are produced, vs. only 80% of proteins with unknown function. The *A. phagocytophilum* genome also includes many repetitive elements (12.7% of the entire HZ genome sequence), containing 113 copies of the *msp2/p44* gene and many variable number tandem repeat (VNTR) sequences (Dunning Hotopp et al., 2006; Barbet et al., 2013; Dugat et al., 2014a,b).

### Tools for studying the genetic diversity of *A. phagocytophilum*

#### Pulsed-field gel electrophoresis (PFGE)

For decades, PFGE was considered the gold standard for epidemiological studies of clinically important bacteria, which is still the case for some species (Sabat et al., 2013). For this method, restriction enzymes cleave total genomic DNA at infrequently occurring restriction sites, and the resulting DNA fragments are then separated on an agarose gel using an alternating electric field.

Dumler et al. developed a PFGE approach for *A. phagocytophilum* (Dumler et al., 2003). However, their method could not discriminate between strains isolated from different animal species, and consequently is not appropriate for epidemiological studies. This technique is laborious, time consuming, and requires specific expertise, and because molecular tools have simultaneously been improved and diversified, becoming both faster and more user friendly, these new approaches have become widely adopted.

#### Single locus sequence typing (SLST)

SLST was the most common molecular approach for studying *A. phagocytophilum* genetic diversity, and is still often used. The *groESL* operon, 16S RNA locus, *ankA*, and *msp2* genes are the most frequently utilized markers.

##### 16S RNA locus

As previously stated, Massung *et al*. described six *A. phagocytophilum* variants (including Ap-V1 and Ap-ha) in the USA, by sequencing the 5′ region of the 16S RNA locus (Massung et al., 2002), and in 2011, 15 variants were defined using this sequence (Rar and Golovljova, 2011). This method can discriminate the human pathogenic variant Ap-ha from other non-pathogenic variants (Massung et al., 2002). However, the authors emphasized this marker's poor resolution, and recommended using other genes to study *A. phagocytophilum* genetic diversity.

In Europe, using the 16S RNA locus, several authors clearly distinguished variants infecting red deer from the those infecting roe deer (Petrovec et al., 2002; Zeman and Pecha, 2008). However, this conclusion was challenged by other studies, where results with this marker did not confirm any host species segregation for *A. phagocytophilum* variants, even including those variants infecting roe deer and red deer (Scharf et al., 2011; Silaghi et al., 2011a; Majazki et al., 2013). Some 16S RNA variants seem to infect wild and domestic ruminants more frequently, whereas other variants have been detected more often in humans, horses and dogs (Silaghi et al., 2011a,b). For this reason, in contrast to the USA, this locus does not seem appropriate for determining if one or many ecotype(s)^6^ circulate in European wild ruminants vs. other host species (Bown et al., 2007; Silaghi et al., 2011a; Overzier et al., 2013). Finally, 16S RNA data is unable to distinguish variants according to their geographical origins (Casey et al., 2004).

Even if some conclusions can be drawn using the 16S RNA locus, it does not have the required level of discrimination, such that markers generating more detailed information are essential for studying *A. phagocytophilum* diversity (Huhn et al., 2014).

##### The groESL operon

The *groESL* operon spans a region covering two genes encoding the GroES and GroEL chaperone proteins, separated by an intergenic region. In Europe, variations in *groESL* can differentiate variants infecting roe deer from those infecting others animals (Rymaszewska, 2008; Silaghi et al., 2011a,b). However, in Austria, other groups detected only one *groESL* variant infecting both red and roe deer (Polin et al., 2004). Using *groEL* sequences, Jahfari et al. described four *A. phagocytophilum* clusters circulating in Europe (Jahfari et al., 2014). The first included all 34 human variants and 209 *A. phagocytophilum* variants obtained from animals (including both wild and domestic animals). The second cluster grouped the majority of roe deer variants (66/72), several sheep variants (5/29), red deer (2/47), and rodent variants (3/30). The third cluster exclusively included rodent variants (27/30), whereas the fourth was only composed of eight *A. phagocytophilum* variants obtained from birds. According to this study, *groESL* could possibly discriminate between those variants isolated from birds and rodents, but not variants originating from other animals.

Also using the gro*ESL* operon, Sumner et al. identified variants circulating in the USA that originated from Europe (Sumner et al., 1997). However, this conclusion could not be generalized for all variants, as only three European strains and five American strains were screened. In another study, Alberti et al. described two clusters: the first grouped pathogenic variants and variants of unknown pathogenicity circulating in both the USA and Europe, whereas the second only included European variants of unknown pathogenicity (Alberti et al., 2005). Within the first cluster, two subclusters were also identified, grouping all variants isolated from the USA and Sardinia on one hand, and a subset of European variants on the other. However, Jahfari et al. could not discriminate variants originating from different European areas using the *groESL* operon (Jahfari et al., 2014).

Thus, the *groESL* operon can distinguish variants of different pathogenicity or geographic origin better than the 16S RNA locus, but further information is required for clearer discrimination of variants and ecotypes.

##### ankA gene

*ankA* encodes an ankyrin repeat protein involved in host cell transcription regulation, which, when sequenced, is able to distinguish five *A. phagocytophilum* variant clusters, relating to the animal host. It must be noted that the majority of samples for the two studies cited here were collected in Europe, except for human variants originating from the USA (Scharf et al., 2011; Majazki et al., 2013). The first cluster mostly contained variants obtained from humans, dogs, cats, and horses, with several variants obtained from domestic and wild ruminants. Within this group, European variants and American human variants belonged to separate subgroups (Rar and Golovljova, 2011). The second cluster included variants isolated from roe and red deer. The third contained variants from domestic (cattle and sheep) as well as wild ruminants (including red and roe deer). The fourth cluster exclusively grouped those variants isolated from roe deer, whereas the fifth was exclusively composed of rodent variants.

Sequencing *ankA* distinguishes variants according to their animal hosts, therefore although this method could be used for phylogenic analysis, it may not have the required level of discrimination for epidemiological studies, such that its applicability remains to be determined.

##### Msp2 gene

Msp2 is an *A. phagocytophilum* surface protein, belonging to the OMP-1/MSP2/P44 superfamily. *msp2* comprises two conserved sequences flanking a hypervariable region, which is sequenced for *A. phagocytophilum* phylogenetic analysis. Using this hypervariable region, one study distinguished European from American variants (Silaghi et al., 2011b). However, horses were the only animal hosts common to these two continents. For this reason, the observed clustering could be linked to the animal host species, rather than to the geographical origin, as suggested by De La Fuente et al. (2005). Before drawing any definite conclusions, larger sample numbers obtained from various American and European animal host species will have to be tested.

Thus, the SLST method can potentially discriminate variants according to their geographical or animal host origins. However, current markers cannot reveal the full genetic diversity of *A. phagocytophilum*. Moreover, depending on the locus used, contradictory results have been obtained. For example, isolates from roe deer and domestic ruminants (sheep and cattle) belonged to different clusters when based on *ankA* gene phylogeny (Scharf et al., 2011), whereas examining the *groEL* locus showed that isolates from domestic ruminants (goats), belonged to the same cluster as those from roe deer (Silaghi et al., 2011a). In order to at least partly solve these problems, multilocus strategies should be employed.

#### Multilocus sequence analysis

##### Multilocus sequence typing (MLST)

The MLST method is based on the PCR amplification and sequencing of several loci. These loci generally consist of seven housekeeping genes, but their functions and numbers can vary according to the bacterial species for which the techniques are developed. For a given locus, each nucleotide sequence obtained corresponds to an allele. The combination of alleles for each locus, also named as a profile, can determine the sequence type (ST) of the microorganism in question. Because housekeeping genes have a rather slow molecular clock, and thus can represent excellent phylogenetic markers if they have been properly selected, MLST has been increasingly recognized as the gold standard phylogenetic method, at least for bacteria with clonal evolution. Various MLST techniques have been developed for *A. phagocytophilum*. Here, we will focus on the two most recent, both developed in 2014 (Chastagner et al., 2014; Huhn et al., 2014). These two techniques have produced results consistent with phylogenies built using 16S RNA and *ankA* loci, and have improved discriminatory power, as could be expected from a technique based on several genes.

The MLST method developed by Chastagner et al. was used to type 160 *A. phagocytophilum*-infected samples obtained from France: 104 cattle samples, 40 roe deer, 13 horse, and 3 dog samples (Chastagner et al., 2014). Interpretable results were obtained for 88 samples (55% typeability). Three clonal complexes were observed. The first only included cattle variants, the second comprised variants isolated from cattle, horses, and dogs, and the third comprised all variants sampled from roe deer and three *A. phagocytophilum* obtained from cattle. The authors concluded that three *A. phagocytophilum* epidemiological cycles exist in French cattle. However, as some potentially important host species, such as red deer, were not included in their analysis, additional studies are needed before drawing definite conclusions.

The second MLST technique, developed by Huhn et al., was used for typing 383 *A. phagocytophilum*-positive samples, obtained from various animal species (Huhn et al., 2014). Interpretable results were obtained for 284 samples (74% typeability). For other samples, double peaks were observed, probably due to sample co-infection by different *A. phagocytophilum* variants. One major clonal complex was detected, which contained the majority of European *A. phagocytophilum* obtained from humans, dogs, and horses. Wild boar and hedgehog variants were also included within this clonal complex, suggesting that they may serve as reservoir hosts for *A. phagocytophilum* capable of infecting humans, horses and dogs in Europe. Variants infecting roe deer and voles were located in another clonal complex, indicating that these animals are involved in (an)other *A. phagocytophilum* epidemiological cycle(s). However, 54% of obtained sequence types did not belong to any of the defined clonal complexes, indicating that the *A. phagocytophilum* population structure could be semi-clonal, raising questions about the reliability of MLST results for this bacterium (Huhn et al., 2014).

##### Multiple locus variable number tandem repeat (VNTR) analysis (MLVA)

The MLVA method is based on PCR amplification of different VNTR. VNTR are mini- or micro-satellite DNA sequences repeated in tandem. The principle of the MLVA method is that depending on the isolate, each VNTR may have a variable number of repeats, thus permitting isolate discrimination. After PCR amplification of a given VNTR, its length is determined by agarose gel migration, or by capillary electrophoresis, and the exact number of repeats is then calculated. Using multiple VNTR increases the discriminatory power of the technique.

Two MLVA techniques for *A. phagocytophilum* typing have been developed to date, the first was developed by Bown et al. (2007). It was based on intergenic microsatellite VNTR, but paradoxically, was too discriminatory for epidemiological studies. The 20 strains studied only shared a few alleles at each of the four loci. For this reason, their technique does not seem well adapted to studying either *A. phagocytophilum* transmission between species, or the circulation of isolates within host populations such as domestic ruminant herds.

The second MLVA technique was developed by Dugat et al. (2014a). In contrast to the first system, this method is based on intragenic minisatellite VNTR, which are theoretically less variable than intergenic microsatellite VNTR. In this study, *A. phagocytophilum* sampled from different cattle herds showed higher diversity than those variants sampled from the same herd, emphasizing the concordance of the information provided by this technique with epidemiological contexts. This technique also identified at least two separate *A. phagocytophilum* clusters based on their hosts: red deer and domestic ruminant variants on one hand, vs. roe deer variants on the other. Epidemiologically unlinked horse variants also grouped together in their own cluster, but only two samples were tested. The authors conclude that at least two *A. phagocytophilum* epidemiological cycles exist in France: the first involving red deer as reservoir hosts, and domestic ruminants as either accidental or longer-term hosts, whereas the second might involve roe deer as reservoir hosts.

##### *A. phagocytophilum* genetic diversity studies using whole genome sequencing

The dramatic cost reduction of whole genome sequencing technologies has enabled their use across a wide range of bacterial genome sequencing projects (Schuster, 2008; MacLean et al., 2009; Fournier et al., 2014). They can now even be used for routine medical investigations in bacteriology (Bertelli and Greub, 2013; Firth and Lipkin, 2013; Fournier et al., 2014). Here we review the current state of the art and future directions for *A. phagocytophilum* genomic studies.

##### Current state of the art

Twenty *A. phagocytophilum* genomes have been sequenced to date, 10 of which were released in March 2015 but without reference to any publication at the time of writing (Dunning Hotopp et al., 2006; Barbet et al., 2013; Dugat et al., 2014b; National Center for Biotechnology Information, 2015). Indeed, the few currently available genomes are far from being representative of global *A. phagocytophilum* diversity. Sixteen genomes are from American strains, whereas only four are from European strains (Table 3). Moreover, with the exception of human variants, only between one and three genomes are available per sampled host species. Thus, neither can these genomes represent the diversity of strains infecting these hosts. Finally, genomes from strains infecting hosts that could play key roles in *A. phagocytophilum* epidemiological cycles, such as roe or red deer, have not yet been released.

**Table 3:** *****A. phagocytophilum*** genomes currently available in public databases**.

**Strain/variant**	**Host**	**Geographical area**	**Size (Mb)**	Number of genes^*^	**Level of assembly**	**References**
HZ	Human (*Homo sapiens*)	USA, New York state	1.47	1411	Close genome	Dunning Hotopp et al., 2006
HZ2	Human (*Homo sapiens*)	USA, New York state	1.48	1141	Close genome	Barbet et al., 2013
ApNYW	Human (*Homo sapiens*)	USA, New York state	1.5	1635	16 scaffolds	National Center for Biotechnology Information, 2015
ApWI1	Human (*Homo sapiens*)	USA, Wisconsin	1.5	1589	1 scaffold	National Center for Biotechnology Information, 2015
HGE1	Human (*Homo sapiens*)	USA, Minnesota	1.47	1188	2 scaffolds	Barbet et al., 2013
HGE2	Human (*Homo sapiens*)	USA, Minnesota	1.48	1548	1 scaffold	National Center for Biotechnology Information, 2015
NCH-1	Human (*Homo sapiens*)	USA, Nantucket	1.5	1646	15 scaffolds	National Center for Biotechnology Information, 2015
Webster	Human (*Homo sapiens*)	USA, Wisconsin	1.48	1570	1 scaffold	National Center for Biotechnology Information, 2015
Dog2	Dog (*Canis lupus familiaris*)	USA, Minnesota	1.47	1148	1 scaffold	Barbet et al., 2013
ApMUC09	Dog (*Canis lupus familiaris*)	Europe, Netherlands	1.52	1675	1 scaffold	National Center for Biotechnology Information, 2015
ApNP	Dog (*Canis lupus familiaris*)	Europe, Austria	1.52	1827	1 scaffold	National Center for Biotechnology Information, 2015
Annie	Horse (*Equus caballus*)	USA, Minnesota	1.52	1642	15 scaffolds	National Center for Biotechnology Information, 2015
MRK	Horse (*Equus caballus*)	USA, California	1.48	1155	9 scaffolds	Barbet et al., 2013
BOV_10-179	Cow (*Bos taurus*)	Europe, France	1.37	1041	169 scaffolds	Dugat et al., 2014b
Norway Variant 2	Sheep (*Ovis aries*)	Europe, Norway	1.52	1174	23 scaffolds	Barbet et al., 2013
JM	*Zapus hudsonius*	USA, Minnesota	1.48	1140	Close genome	Barbet et al., 2013
CR1007	*Tamias striatus*	USA, Minnesota	1.5	1554	4 scaffolds	National Center for Biotechnology Information, 2015
CRT38	*Ixodes scapularis*	USA, Minnesota	1.51	1203	2 scaffolds	Barbet et al., 2013
CRT35	*Ixodes scapularis*	USA, Minnesota	1.45	1148	25 scaffolds	Barbet et al., 2013
CRT53-1	*Ixodes scapularis*	USA, Minnesota	1.57	1655	45 scaffolds	National Center for Biotechnology Information, 2015

* *Genes include complete CDS, rRNA, tRNA and pseudogenes*.

In summary, whereas whole genome sequencing is a powerful tool for studying *A. phagocytophilum* genetic diversity, there are too few published genomes to fully exploit their potential, especially when considering the low number of genomes available for each investigated animal species. More genomes sequenced from strains or samples originating from various animal hosts and geographical areas are understandably needed.

##### Perspectives for *A. phagocytophilum* genomic studies

The difficulty of cultivating *Anaplasma sp*. can be a critical barrier for accessing genomic sequences. In order to circumvent this difficulty, some authors have attempted to sequence *Anaplasma sp*. genomes without the intervening culturing steps (Dark et al., 2012; Dugat et al., 2014b).

In 2012, Dark et al. sequenced the genome of *Anaplasma marginale* Florida and St. Maries strains directly from infected cattle blood samples (Dark et al., 2012). They performed whole genome amplification using the Phi29 DNA polymerase, followed by *A. marginale* genome sequencing. This approach was probably successful because *A. marginale* multiplies in anucleate erythrocytes. In nucleated cells, host DNA would inevitably contaminate the bacterial genomic amplification reaction. In the same study, Dark et al. also successfully sequenced the genome of the *A. phagocytophilum* HZ strain, cultivated in HL-60 cells (Dark et al., 2012). A bacterial purifying step was still required with this method, and its efficiency for sequencing *A. phagocytophilum* genomes directly from field samples is yet to be explored.

Another strategy without the need for bacterial isolation or purification, was proposed by Dugat et al. (2014b). Using a whole genome capture approach, they sequenced the genome of *A. phagocytophilum* directly from an infected cattle blood sample. This method enriched *A. phagocytophilum* DNA 197-fold in the field sample, permitting successful sequencing. This is the first reported *A. phagocytophilum* whole genome sequencing performed directly from a field sample. The 1.37 Mb BOV-10_179 *A. phagocytophilum* genome obtained was composed of 169 scaffolds, compared to 56 in the approach used by Dark et al. (2012), and included 1281 coding DNA sequences. As the study of Dark et al. resequenced the HZ strain genome (first sequenced by Dunning Hotopp et al., 2006) it is not surprising that the number of scaffolds obtained was lower than the *de novo* sequencing project of the BOV-10_179 genome.

To capture the European bovine BOV-10_179 genome, Dugat et al. designed capture probes using the American human HZ strain genome, which was the only available *A. phagocytophilum* genome at the time. For this reason, and also due to the differences between human and ruminant strains (and potentially between American and European strains) (Barbet et al., 2013), it is plausible that at least some specific BOV-10_179 sequences have not been captured. However, is it interesting to note that the capture probes also captured flanking regions around target sequences, which could minimize this phenomenon. Using the 20 *A. phagocytophilum* genomes that are currently available, new capture probes could be designed which would better represent *A. phagocytophilum* genetic diversity, facilitating the capture of a wider range of *A. phagocytophilum* genome sequences. Finally, according to their results, the authors concluded that it should be relatively simple to sequence several *A. phagocytophilum* genomes via multiplexing, without compromising the results in terms of coverage (Dugat et al., 2014b). Thus, whole genome capture is a promising tool for studying *A. phagocytophilum* genetic diversity.

## Conclusion

In conclusion, distinct *A. phagocytophilum* ecotypes appear to be adapted to different vertebrate hosts and/or vectors. As a result, *A. phagocytophilum* ecotypes could circulate within several different epidemiological cycles, which could be independent or interconnected. This adds additional complexity to the puzzle of *A. phagocytophilum* epidemiology, and alongside the lack of sufficiently accurate or well-adapted molecular tools, explains why these epidemiological cycles have not yet been completely elucidated. Indeed, until just a few years ago, single locus sequence typing techniques were used for studying both *A. phagocytophilum* epidemiology and phylogeny. During that time, various multilocus typing techniques have been developed. These techniques are an impressive stride forward in improving knowledge of *A. phagocytophilum* diversity. In the near future, whole genome sequencing will enable access to the entire genetic diversity of *A. phagocytophilum*, and will also facilitate the development of whole genome typing techniques such as single-nucleotide polymorphism approaches or whole genome MLST. These techniques will be invaluable in unlocking the black box of *A. phagocytophilum* diversity, and promoting continual cross-talk between the laboratory and the field.

### Conflict of interest statement

The authors declare that the research was conducted in the absence of any commercial or financial relationships that could be construed as a potential conflict of interest.

## Footnotes

^1^Centers for Disease Control and Prevention, Statistics and Epidemiology of Anaplasmosis. Available online at: http://www.cdc.gov/anaplasmosis/stats/ (Accessed April 23, 2014).

^2^A genetic variant is defined here as a population identified within a bacterial species using one or more genetic markers (such as the 16S RNA locus), without any obligatory links to phenotypic variations. This subdivision depends on the marker used, and thus on the technique performed.

^3^A vector is defined here as an arthropod which transmits a pathogen from a receptive host (i.e., able to be infected by the pathogen and permits pathogen multiplication without developing symptoms) and/or a susceptible host (i.e., which can develop symptoms), to another receptive and/or susceptible host.

^4^The transmission of a pathogen by co-feeding is defined by Jones et al. as “an exchange of a pathogen between infected and uninfected arthropod vectors feeding simultaneously on a host in the absence of systemic infection” (Jones et al., 1987).

^5^In this review, for simplification purposes, the term “prevalence” refers to studies in which the prevalence was determined by detection of *A*.*phagocytophilum* DNA. The term “seroprevalence” is used when the prevalence was estimated through detection of specific antibodies.

^6^For *A*.*phagocytophilum*, an ecotype is defined as a bacterial population sharing the same ecological niche.

## Abbreviations

BGA: Bovine granulocytic anaplasmosis
CDC: Centers for Disease Control
EGA: Equine granulocytic anaplasmosis
HGA: Human granulocytic anaplasmosis
MLST: Multilocus sequence typing
MLVA: Multiple locus VNTR analysis
OGA: Ovine granulocytic anaplasmosis
PFGE: Pulsed-field gel electrophoresis
SLST: Single locus sequence typing
ST: Sequence type
TBF: Tick-borne fever
VNTR: Variable number tandem repeat.
